# Resistant ovary syndrome: Pathogenesis and management strategies

**DOI:** 10.3389/fmed.2022.1030004

**Published:** 2022-10-19

**Authors:** Zhenni Mu, Sinan Shen, Lei Lei

**Affiliations:** College of Integrated Traditional Chinese and Western Medicine, Hunan University of Chinese Medicine, Changsha, China

**Keywords:** resistant ovary syndrome, etiology, pathogenesis, infertility, management strategies

## Abstract

Resistant ovary syndrome (ROS) is a rare and difficult gynecological endocrine disorder that poses a serious risk to women’s reproductive health. The clinical features are normal sex characteristics, regular female karyotype, and usual ovarian reserve, but elevated endogenous gonadotropin levels and low estrogen levels with primary or secondary amenorrhea. Although there have been many case reports of the disease over the past 50 years, the pathogenesis of the disease is still poorly understood, and there are still no effective clinical management strategies. In this review, we have collected all the current reports on ROS and summarized the pathogenesis and treatment strategies for this disease, intending to provide some clinical references for the management and treatment of this group of patients and provide the foothold for future studies.

## Introduction

Resistant ovary syndrome (ROS), also known as ovarian insensitivity syndrome or Savage syndrome, is a rare gynecological condition with heterogeneous etiology that was first identified and named by de Moraes-Ruehsen and Jones (1).

Patients present clinically with primary or secondary amenorrhea before the age of 30 years of age, with a low response to exogenous gonadotropins and biochemical tests suggesting elevated endogenous gonadotropin levels and low estrogen levels. Moreover, ROS patients present with normal karyotype and normal ovarian reserve, i.e., normal Anti-Mullerian Hormone (AMH) and inhibin B (INB) levels, and with normal numbers of small follicles on vaginal ultrasound and laparoscopic ovarian histology (2–4).

According to the World Health Organization (WHO) classification (5), ROS belongs to type III amenorrhea characterized by hypergonadotropic hypogonadism (6, 7). It is often difficult to distinguish clinically from primary ovarian insufficiency (POI) or premature ovarian failure (POF) and is considered a subtype of POI or POF.

## Etiology and pathogenesis

Current research on ROS is still in its preliminary stage. Given its low prevalence, it has predominantly been reported as scattered cases, and no large sample-size studies have been conducted. Studies on the mechanism have mostly focused on gonadotropin pre-receptor and partial receptor levels. Deficiency of follicle-stimulating growth factors, mutations in the follicle-stimulating hormone (FSH) receptor or beta subunit, abnormal gonadotropin signaling, and autoimmune problems are potential causes of this disorder. It has been established that the mechanism of ROS involves the failure of gonadotropins to act effectively on the follicles. Accordingly, the follicles fail to develop normally and stagnate in a resting state.

### Inactive mutations of follicle-stimulating hormone

**FIGURE 1:**
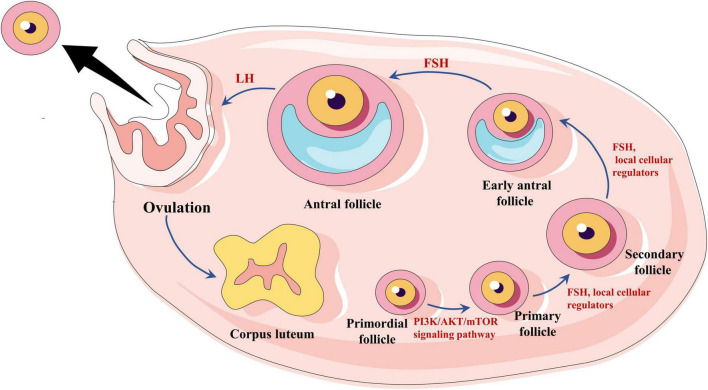
Hormonal regulation of follicle growth and expulsion. Primordial follicles are regulated by the PI3K/AKT/mTOR signaling pathway to a primary stage of development, followed by the development of sinus follicles by the action of follicle-stimulating hormone (FSH) and local cellular regulators. Eventually, the oocyte matures under the influence of LH, and ovulation occurs.

Current evidence suggests that primordial follicles develop to the primary stage mediated by the PI3K/AKT/mTOR signaling pathway (8) (initial recruitment), while most primordial follicles remain inhibited until they receive activation signals. Once growth begins, the primordial follicles develop into sinus follicles in response to local cellular regulators in the ovary, such as keratinocyte growth factor (KGF) (9), platelet-derived growth factor (PDGF) (10), basic fibroblast growth factor (bFGF) (11), and so on. Although most early sinus follicles undergo atresia, at least one sinus follicle will progress to preovulation (circulating recruitment) under pituitary FSH and luteinizing hormone (LH) stimulation (12). During the later stages of follicular development, FSH provides the primary stimulus (13, 14; Figure 1). Intriguingly, inactivating FSH mutations result in many sinus follicles developing without the support of endogenous FSH and failing to develop into dominant follicles, remaining in the primary stage. Clinically, several small follicles without dominant follicles can be observed under vaginal B-ultrasound.

### Abnormalities in the regulation of follicle-stimulating factors

**FIGURE 2:**
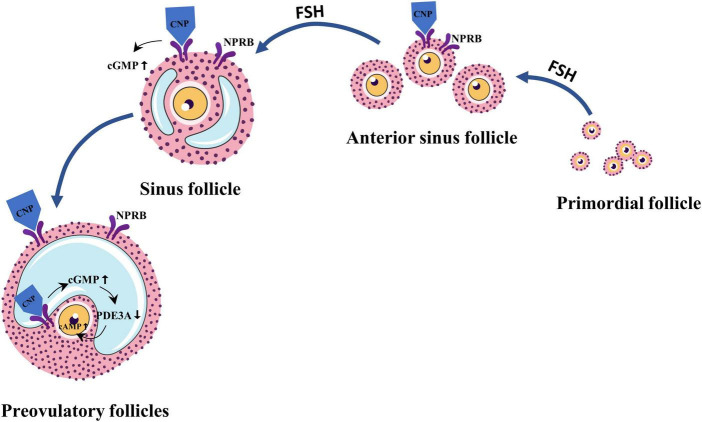
C-type natriuretic peptide (CNP) is an important follicle growth regulator secreted by granulosa cells in secondary and sinus follicles in response to follicle-stimulating hormone (FSH) stimulation. Cyclic guanosine monophosphate (cGMP) and cyclic phosphate (cAMP) levels are regulated by CNP in combination with natriuretic peptide receptor-B (NPRB), thereby regulating follicle development and maturation.

Besides FSH, C-type natriuretic peptide (CNP) is a follicle-stimulating factor that has been identified in recent years (12, 15). CNP belongs to the natriuretic peptide family and is characterized by a highly conserved 17-membered ring structure formed by intramolecular disulfide bonds (16). It was found that the precursor protein natriuretic peptide precursor C (NPPC) of CNP and its cognate receptor natriuretic peptide receptor-B (NPRB) were expressed in sinus follicles and preovulatory follicles (17). CNP is produced by the sinus follicle and binds to its receptor to stimulate follicle development by activating the guanylate cyclase coupled receptor to produce cyclic guanosine monophosphate (cGMP) (18, 19). In addition, in the preovulatory follicle, CNP stimulates cGMP production by activating NPRB, which is expressed by perivitelline and oocyte-associated cumulus cells (CCs) and diffuses into oocytes *via* gap junctions (20). In oocytes, cGMP inhibits phosphodiesterase 3A, thereby preventing cAMP hydrolysis (20). Overwhelming evidence suggests that adenosine 3′, 5′-cyclic phosphate (cAMP) is a second messenger in various central cellular functions. In the ovaries, cAMP regulates ovulation, enhances primordial follicle growth, and provides a key signal for gonadotropin action on the gonads. High levels of cAMP can promote growth in primordial follicles (21) and inhibit oocyte maturation (20). Sato et al. (18) reported that daily injections of CNP to infant mice could promote follicle growth, and ovulation was successfully induced by gonadotropins. This was also demonstrated by *in vitro* studies (22). This finding suggests that CNP is essential for follicular growth and development. Regional follicular growth and development in ROS patients may be related to the abnormal regulation of this factor (Figure 2).

### Mutations in the follicle-stimulating hormone receptor gene

The interaction of FSH with its receptor is critical for follicle development and maturation. Interestingly, any variation in the FSHR genotype may alter the receptor’s ability to bind FSH and induce downstream signaling pathways. Inactivating mutations in the FSHR gene have been associated with loss of ovarian function in women, and these mutations lead to impaired receptor function (23). Abel et al. (24) found that follicles developed to the antral follicle stage in mice with disrupted FSHR gene, suggesting that the FSHR defect does not affect the development of the antral follicle, accounting for a large number of primordial follicles in the ovary of ROS patients with no sinus follicle development.

It is well-established that mutations in the FSHR are rare, and only 25 loss-of-function mutations in FSHR have been found in women with ovarian dysgenesis, primary amenorrhea, and secondary amenorrhea (Table 1). Of these, the C 566 T mutation is only seen in Finns, suggesting a possible interethnic difference (25). Mutations in the FSHR gene are reportedly rare in UK women with ROS (26). However, most studies had small sample sizes and did not provide robust evidence that FSHR gene polymorphisms have pathophysiological significance in ovarian function.

**TABLE 1:** Inactivating mutations of follicle-stimulating hormone receptor (FSHR) previously reported in women with ovarian dysgenesis, primary amenorrhea, and secondary amenorrhea.

No.	Nucleotide change (exon number)	Amino acid change	Phenotype of subjects	References
1	c.566C>T (exon 7)	p.Ala189Val	Primary amenorrhea with increased serum FSH levels	(27)
2	c.662T>G (exon 8)	p.Val221Gly	Primary amenorrhea	(28)
3	c.671A>T (exon 7) c.1801C>G (exon 10)	p.Asp224Val p.Leu601Val	Primary amenorrhea with increased serum FSH levels	(23)
4	c.1043C>G (exon 10)	p.Pro348Arg	Primary amenorrhea with increased serum FSH levels	(29)
5	c.1255G>A (exon 10)	p.Ala419V al	Primary amenorrhea with increased serum FSH levels	(30)
6	c.1555C>A (exon 10)	p.Pro519Thr	Primary ovarian failure with increased FSH levels	(31)
7	c.1760C>A (exon 10)	p.Pro587His	Primary amenorrhea	(32)
8	c.1723C>T (exon 10)	p.Ala575Val	Primary amenorrhea with hypergonadotropic hypogonadism	(25)
9	c.175C>T (exon 2)	p.Arg59Term	Primary ovarian insufficiency with increased serum FSH levels	(33)
10	c.1222G>T (exon 10)	p.Asp408Tyr	Primary amenorrhea with increased serum FSH levels	(34)
11	c.1253T>G (exon 10)	p.Ile418Ser	Primary ovarian failure and hypergonadotropic hypogonadism	(35)
12	c.1298C>A (exon 10)	p.Ala433Asp	Hypergonadotropic hypogonadism	(36)
13	c.419delA c.1510C>T c.44G>A (exons 1 and 2)	p.Lys140Argfs*16 p.Pro504Ser p.Gly15Asp	Primary ovarian insufficiency with resistant ovary syndrome	(37)
14	I423T (exon 10)	–	Primary amenorrhea with primary ovarian failure	(38)
15	c.479C>T (exon 6) c.1717C>T (exon 10)	p.Ile160Thr p.Arg573Cys	Secondary amenorrhea with increased serum FSH levels	(39, 40)
16	c.182T>A (exon 2) c.2062C>A (exon 10)	p.Ile61Asn p.Pro688Thr	Secondary amenorrhea with resistant ovary syndrome	(41)
17	c.793A>G (exon 9) c.1789C>A (exon 10)	p.M265V p.L597I	Secondary amenorrhea with premature ovarian insufficiency	(42)
18	c.646G>A (exon 8) c.1313C>T (exon 10)	p.Gly216Arg p.Thr438Ile	Secondary amenorrhea with premature ovarian insufficiency	(43)

### Abnormal regulation of granulocyte proliferation factors

At least three oocyte-derived factors have been shown to promote the growth of granulosa cells, including R-spondin2, growth differentiation factor 9 (GDF9), and bone morphogenetic protein 15 (BMP15) (12). Studies in mice have revealed that the transcripts of R-spondin2 are only present in primary oocytes and oocytes of larger follicles but not in the initiating follicles (44). The decrease of R-spondin2 level may lead to failure of follicle development in the late reproductive phase (12), which may contribute to the fact that in ROS patients, during repeated ovulation stimulation, even when follicles develop, they reach atresia occurs before dominance. If similar R-spondin2 effects are identified in humans, R-spondin agonists could provide a new therapeutic approach for infertile women (12).

In addition to R-spondin2, GDF9 and BMP15 are local factors produced by oocytes that stimulate follicle development. They are members of the TGF-β superfamily of cystine junctional proteins (45) and bind to receptor serine kinases (RSK) to stimulate downstream signaling (46). Both factors bind to type II RSK BMP receptor II (47) and recruit type I RSK for GDF9, and BMP15 to regulate downstream SMAD proteins in granulosa cells. Current evidence suggests that GDF9 treatment enhances the growth and differentiation of cultured prezygotic follicles (48). *In vivo*, treatment with GDF9 promotes the development of primordial follicles to primary and antral follicles (49). GDF9 also has antiapoptotic effects during early sinus follicle development (50). In the ovaries of ROS patients, follicles are often present in the primordial state, which may be associated with a lack of or abnormal regulation of the GDF9 factor.

### Immunity-related issues

Many studies have shown that the pathogenesis of ROS is related to immune factors. Most studies found that patients with ROS may have autoantibodies against FSHR that block the ovarian response to gonadotropin stimulation (51–55). In this respect, Rogenhofer et al. (6) identified antibodies against human menopausal gonadotropin (HMG) that were not recombinant-FSH (re-FSH) in a patient with ROS. It has also been found that IgG can block DNA synthesis of granulosa cells stimulated by FSH in ROS patients (56). In addition, Chiauzzi et al. (52) found immunoglobulin (Ig-FSHR) in the blood of patients with ROS that inhibited the binding of FSH to its receptor by detecting the level of circulating immunoglobulin in patients with ROS. Escobar et al. (57) reported a case of myasthenia gravis with ROS whereby a substance behaving like gamma globulins could inhibit binding to FSH-specific receptors in an *in vitro* system.

In contrast, Starup and Pedersen (58) found no circulating gonadotropin antibodies in a 21 years-old woman with ROS. Consistently, Board et al. (59) found no autoimmune antibodies in ROS patients. Moreover, recent case reports of ROS in which patients were examined for immune disorders showed no signs of immune disorders (anti-nuclear antibodies, antiphospholipid antibodies, lupus antibodies, antibodies to semicarbazide, adrenocortical autoantibodies, steroid cell autoantibodies, serum 21-hydroxylase, 17-hydroxylase, and P450 side-chain cleavage enzyme autoantibodies were all negative) (60–62).

## Management strategy

**FIGURE 3:**
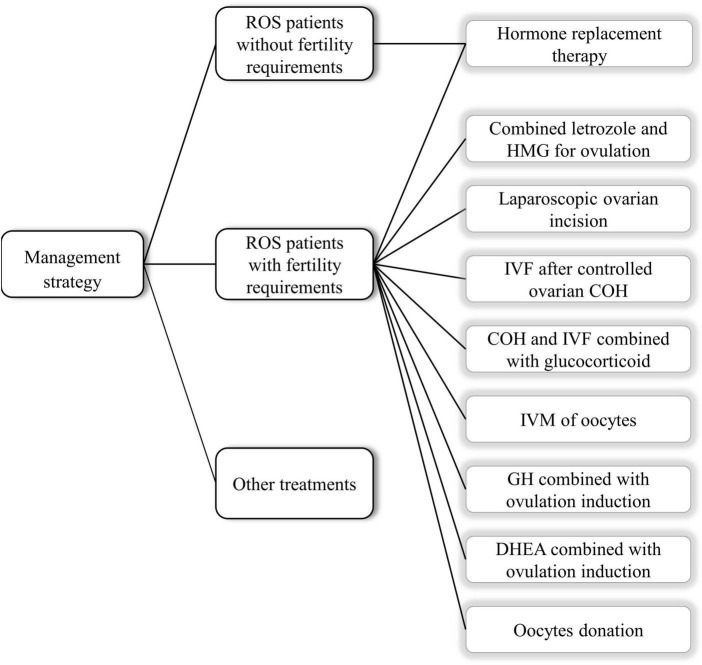
The specific clinical treatments of resistant ovary syndrome (ROS) patients.

The clinical treatment varies for women of different ages, with various complaints and other clinical manifestations. For girls during puberty, the main aim is to promote the development of secondary sexual characteristics, maintain normal menstrual flow and protect the function of the ovaries, mainly using hormone supplementation; for women of childbearing age without fertility requirements, the basic principle of treatment is to provide physiological supplementation and prevent diseases in other systems caused by hormone deficiency. In contrast, assisted reproductive techniques such as ovulation promotion and *in vitro* culture of immature oocytes can be performed for women of childbearing age with fertility requirements. All specific treatment options suggested are based on case reports (Figure 3).

### Management strategy of resistant ovary syndrome patients without fertility requirements

#### Hormone replacement therapy

For adolescents or women of childbearing age who do not require fertility, treatment of ROS usually begins with hormone replacement therapy (HRT) to maintain normal menstruation. Estrogen (e.g., estradiol valerate 2–4 mg/d) and progestin (e.g., norgestrel 500 mg/d) are administered for 22–28 days (63, 64). Withdrawal bleeding occurs on days 3–7 after discontinuation, and the next cycle is administered on the 5th day of menstruation, usually for three consecutive cycles. In addition, short-acting oral contraceptive pills (OCP) can be used to establish an artificial menstrual cycle for patients.

### Treatment of resistant ovary syndrome patients with fertility requirements

#### Hormone replacement therapy

For ROS patients with fertility requirements, ovulation induction therapy or other assisted fertility methods are usually performed based on HRT (usually ≥3 months). However, cases of ROS having spontaneous pregnancies and successful live births during or after treatment with HRT have been extensively reported in the literature (63–68).

Mueller et al. (63) reported on a 26 years-old woman with primary infertility with decreased serum FSH and LH levels and increased levels of estradiol, progesterone, and inhibin A during the third month of HRT, leading to spontaneous follicular growth, maturation, and ovulation. One cycle after discontinuation of the drug, there was still spontaneous ovulation of follicles, and the patient successfully conceived. Lim et al. (69) reported a 24 years-old ROS woman with secondary amenorrhea and primary infertility. After 2 months of cyclic estrogen-progestin replacement therapy, insufficient inhibition of FSH and LH was observed. Subsequently, she was prescribed 100 pg of Ethinyl Estradiol daily for 23 consecutive months and successfully conceived.

It is possible that exogenous estrogen suppressed excessive gonadotropins in the body, resulting in increased sensitivity of FSH receptors on follicular membrane cells and increased sensitivity to gonadotropins, allowing follicles to become sensitive to ovulatory drugs or spontaneously ovulate and conceive (70). Although individual cases cannot indicate whether the treatment was effective or occurred by chance, it has been established that approximately 13% of ROS patients can become pregnant spontaneously after low-dose HRT. Accordingly, pregnancy is still possible after HRT treatment in ROS patients (71).

#### Combined letrozole and human menopausal gonadotropin for ovulation

Letrozole is a third-generation aromatase inhibitor, and its peripheral action is key to the successful induction of ovulation in patients with ROS. In the periphery, letrozole blocks the conversion of androgens to estrogens at the ovarian level by inhibiting aromatase activity, leading to a transient accumulation of androgens in the ovary, which in turn stimulates the expression of insulin-like growth factor I and other autocrine and paracrine factors and increases ovarian responsiveness to gonadotropins (72). The combination of HMG and letrozole can maximize its effect and reduce the dosage of HMG. Mu et al. (73) reported the successful induction of dominant follicle development and ovulation in a patient with ROS after ovulation promotion with letrozole combined with HMG, resulting in the live birth of a healthy male infant.

#### Laparoscopic ovarian incision

In recent years, laparoscopy has been widely used in treating gynecological diseases due to its low invasiveness, good surgical field, rapid healing, low risk of infection, low impact on the abdominal organs and high safety profile. LOI is a novel and simple laparoscopic procedure that promotes follicular growth in patients with follicular maturation disorders by mechanical damage to the ovarian cortex (74) to provide the intrinsic stimulation needed for dormant follicles.

Tanaka et al. (75) performed LOI in 11 patients with ROS, followed by controlled ovarian hyperstimulation (COH) for at least 1 year. Four ROS patients became pregnant and delivered three healthy babies with one ongoing pregnancy.

#### Controlled ovarian hyperstimulation and *in vitro* fertilization

Unlike conventional COH, the COH scheme of ROS patients may be slightly different due to the lack of antibodies or receptors in the ovary in ROS patients and there is no clear clinical consensus on this.

Rogenhofer et al. (6) reported a 26 years-old woman with secondary amenorrhea with fertility appeal that was given HRT. On day 20 of the third cycle, Gonadotropin-releasing hormone agonist (GnRH-a) was used for downregulation, and 75IU rec-FSH-β and 225 IU HMG were injected subcutaneously on day 10 of downregulation. Ultrasound examination showed 12 dominant follicles 14 days after treatment. Intracytoplasmic sperm injection (ICSI) was performed after egg removal, and a boy was born naturally after transplantation. The patient received a steady and sustained suppression of gonadotropins, thus increasing ovarian sensitivity, which may be attributed to pituitary downregulation (76).

#### Controlled ovarian hyperstimulation and *in vitro* fertilization combined with glucocorticoids

Glucocorticoids (GC) are extremely important regulatory molecules in the body and play an important role in regulating the development, growth, metabolism and immune function of the body. In clinical practice, they are widely used as anti-inflammatory and immunosuppressive agents (77). Dexamethasone is a long-acting GC widely used in allergic and autoimmune inflammatory diseases. Li et al. (78) reported a case of ROS with serological evidence of antibodies against FSHR, who eventually achieved a live birth after ovarian stimulation combined with dexamethasone treatment. Hydrocortisone is a short-acting GC with anti-inflammatory and anti-allergic effects and is widely used in immune disorders. Riestenberg et al. (79) documented a case of Ig-FSHR-related POI initially resistant to maximal dose gonadotropin stimulation that eventually underwent successful COH and oocyte cryopreservation using short-term high-dose prednisone for transient immunosuppression. The above two cases indicate that GC suppression of abnormal autoimmune antibodies may be used for ROS treatment.

#### *In vitro* maturation of oocytes

*In vitro* maturation is a method of obtaining immature cumulus-oocyte complexes from antral follicles with or without ovulation medication, culturing them *in vitro* under suitable conditions to mature to the MII stage, followed by IVF/ICSI to achieve pregnancy (80). Indications for IVM include cases of ROS with impaired oocyte maturation. Since 2013, several cases of ROS in women successfully conceiving following IVM have been reported clinically (62, 81–87). Galvao et al. (83) reported that nine patients with ROS underwent 24 IVM cycles and found that the live birth rate was 16.7% per started cycle and 33.3% per patient. Therefore, IVM offers a meaningful approach to fertility claims in ROS patients, but it is more costly.

#### Growth hormone combined with ovulation induction

It is widely acknowledged that the reproductive and growth axes often interact, and GH indirectly affects ovarian development and its sensitivity to gonadotropins during puberty through gonadotropins and insulin-like growth factor 1 (IGF-I) (88). Growth hormone deficiency or insufficiency leads to delayed puberty and affects normal ovarian function. Studies in recent years have shown that growth hormone can improve ovarian responsiveness, promote endometrial growth, improve ovulation treatment in patients with low ovarian response, and increase pregnancy rates with cyclic ovulation. However, the use of growth hormone in the promotion of ovulation in patients with ROS warrants further investigation (89–92). Mueller et al. (63) reported a case of ROS in which follicle growth was not successfully induced with growth hormone. There are no reports in the literature on the effectiveness of growth hormones in promoting ovulation in patients with ROS.

#### Dehydroepiandrosterone combined with ovulation induction

Dehydroepiandrosterone (DHEA) is a steroid abundant in human blood circulation (93). It enters the circulation mainly in the form of DHEA sulfate (DHEA-S), which has weak androgenic effects and is converted to testosterone and estradiol in peripheral tissues (94). Interestingly, Zangmo et al. (95) found an increase in the number of oocytes, fertilization rates, and whole embryos in IVF cycles in patients with poor ovarian response to DHEA. Similarly, some studies confirmed that DHEA could optimize the fertility of POI patients and lead to successful pregnancy (96, 97). However, Wong et al. (98) found no benefit of DHEA supplementation on functional improvement in POI patients through a prospective observational study.

#### Oocyte donation

With the development of assisted reproductive technology, the technical challenges of oocyte donation have largely been resolved, but the ethical issues it raises are also a hot topic of the current debate on the technology (99). It is widely thought that donor oocytes can be used for *in vitro* fertilization transplantation only in ROS patients who have not responded well to long-term ovulation-promoting drugs.

### Other treatments

In addition to the above treatments, appropriate calcium and vitamin D supplementation can prevent osteoporosis due to estrogen deficiency in ROS patients (100); appropriate psychological support is also helpful in restoring follicular development and ovulation in ROS patients (101). In addition, Chinese medicine has been reported to assist in treating ROS patients *via* kidney tonification. However, specific treatment effects need to be studied in large sample size studies.

## Conclusion

Research is still ongoing to understand the complex pathogenesis of ROS, given its intricacy. The exact mechanisms remain largely unclear, and currently available approaches are often ineffective. The heterogeneity in the etiology of ROS account for the wide range of individual treatment options, and treatment modalities such as psychological support, artificial cycles, and ovulation promotion do not address the root of the patient’s problem. The poor effectiveness of treatment is often accompanied by the psychological and financial strain of long-term medication on patients. Accordingly, an in-depth understanding of the pathogenesis of the disease is an important prerequisite for studying ROS management strategies. With the development of assisted reproductive technology, new assisted reproductive methods such as IVM, GH, and DHEA addition may be able to solve the fertility problems of this patient population, but more comprehensive and effective management strategies need to be further investigated.

## Author contributions

LL conceived and directed the manuscript writing. ZM reviewed the literature and wrote the manuscript. SS reviewed the literature. All authors contributed to the article and approved the submitted version.
